# Effects of the search technique on the measurement of the change in quality of randomized controlled trials over time in the field of brain injury

**DOI:** 10.1186/1471-2288-5-7

**Published:** 2005-02-07

**Authors:** Mark K Borsody, Chisa Yamada

**Affiliations:** 1 Department of Neurology Northwestern Memorial Hospital Chicago, Illinois 60611 USA; 2 Department of Pathology Albert Einstein University Bronx, New York 10463 USA

## Abstract

**Background:**

To determine if the search technique that is used to sample randomized controlled trial (RCT) manuscripts from a field of medical science can influence the measurement of the change in quality over time in that field.

**Methods:**

RCT manuscripts in the field of brain injury were identified using two readily-available search techniques: (1) a PubMed MEDLINE search, and (2) the Cochrane Injuries Group (CIG) trials registry. Seven criteria of quality were assessed in each manuscript and related to the year-of-publication of the RCT manuscripts by regression analysis.

**Results:**

No change in the frequency of reporting of any individual quality criterion was found in the sample of RCT manuscripts identified by the PubMed MEDLINE search. In the RCT manuscripts of the CIG trials registry, three of the seven criteria showed significant or near-significant increases over time.

**Conclusions:**

We demonstrated that measuring the change in quality over time of a sample of RCT manuscripts from the field of brain injury can be greatly affected by the search technique. This poorly recognized factor may make measurements of the change in RCT quality over time within a given field of medical science unreliable.

## Background

Considerable effort has been directed toward improving randomized controlled trial (RCT) design, execution, and reporting [1-6,14]. Such efforts to define standards of quality for RCTs beg the question: are RCTs improving in quality over time? Many reviews have attempted to answer this question. In general, these reviews measure the presence or absence of several criteria chosen to define quality in a sample of RCT manuscripts that was selected from a parent population of RCT manuscripts. The parent population of RCT manuscripts may be either a field of medical science or a defined part of the medical literature (e.g., RCT manuscripts from a chosen journal). Then, by examining a score of quality as a function of the year-of-publication of the sampled RCT manuscripts, conclusions are drawn as to whether or not quality is changing over time in the parent population of RCTs. If such reviews are to be useful, then, the sample of RCT manuscripts that was chosen for analysis must represent the parent population of RCT manuscripts.

As much as the RCT manuscripts published in a single journal or group of journals would provide a well-defined parent population, the RCT manuscripts from a given field of medical science would be difficult to completely identify. Ultimately no search strategy can claim to identify all manuscripts on a given topic that have been published in every book and journal worldwide. Thus, two search techniques might provide considerably different samples of RCT manuscripts from the same field of medical science depending upon how much and / or what parts of the parent population of RCT manuscripts they can access. The current communication empirically demonstrates this point as a potential pitfall in measuring the change in quality over time of RCT manuscripts sampled from a representative field of medical science.

## Methods

### Criteria of quality

We chose internal validity as a measure of quality according to the definition given by Gehlbach [7], namely that a RCT is internally valid when "within the confines of the study, results appear to be accurate and interpretation of the investigators is supported". We selected criteria of internal validity according to the recommendations of Moher et al. [8]. The relevant points are addressed below.

#### I. Definition of the quality construct

We intended to measure the presence or absence of various criteria of RCT quality as described in the published manuscript. No attempt was made to contact the authors of a manuscript either to clarify the information provided in the manuscript or to gain additional information about a RCT. We acknowledge that relying on the published manuscript in order to assess the quality of a RCT may be biased (1) against well-designed RCTs that were reported in poorly written manuscripts and (2) in favor of poorly-designed RCTs that were reported in well-written manuscripts [9]. Thus, our scoring process ultimately measured the quality of the report of the RCT manuscript, rather than the true methodological quality of the trial as it was conducted. However, attempting to obtain an understanding of the true methodological quality of a RCT in a retrospective manner by contacting the authors of the manuscripts would undoubtedly collect more information on recent RCTs because their authors will be more accessible (i.e., less likely to have relocated, retired, or died). Attempting to contact the authors of manuscripts is rarely successful [10] and, when it is successful, accurate information about the design and conduct of the RCT is not always forthcoming [11,12].

#### II. Definition of the scope of internal validity and identification of quality criteria

Although random allocation and the use of a concurrent control group are the *sine qua non *of the RCT, additional criteria have been so frequently included in their design and execution that they are now commonly considered as part of quality RCTs. Several sources (themselves located by PubMed MEDLINE and bibliography searches) were used to identify such criteria [2,9,13-18]. After forming a composite list of internal validity criteria from these sources, we searched the literature (again by means of PubMed MEDLINE and bibliographies) for instances where the presence or absence of each criterion in a RCT affected the results obtained from the RCT. Thus, we identified criteria that were supported by empirical evidence as measures of RCT quality. We identified six criteria that had predominantly supporting evidence in their favor. Subsequently, allocation concealment was included as a separate quality criteria. The quality criteria, with brief descriptions, are listed in Table 1.

**Table 1:** The quality scale This table lists the criteria of quality that were used to score the RCT manuscripts. Abbreviated definitions for the presence (1 point) or absence (0 points) of each criterion are provided.

1) assessment of the distribution of patient characteristics and prognostic factors between groups	
present	distribution of patient characteristics and prognostic factors assessed without asymmetry between groups
absent	not mentioned; distribution of patient characteristics and prognostic factors assessed with asymmetry noted between groups
2) prevention of the movement of patients between groups after allocation, and the use of intention-to-treat analysis	
present	use of intention-to-treat analysis; no movement of patients between groups confirmed
absent	not mentioned; patients known to change groups before analysis
3) the blinding of the patients to the treatment they received	
present	statements of double-blind present; use of a placebo; statements of the treatments being indistinguishable present; patients not aware of study due to clinical condition
absent	not mentioned; lack of placebo use in control group; readily-distinguishable treatments; blinding breakdown confirmed
4) the blinding of the health care providers to the treatments received by the patients	
present	third-party dispensation of treatments; statements of health care provider blinding present; health care provider identical to outcome observer, and outcome observer is blinded
absent	not mentioned; health care team aware of patient allocation; lack of placebo in control condition; readily-distinguishable treatments; blinding breakdown confirmed
5) the blinding of the outcome observer to the treatment received by the patient	
present	statements of double blind present; objective outcome; use of standardized tests or questionnaires that do not require an outcome observer; subjective principle outcome but outcome observer blinded to treatment; blinded health care providers performing outcome assessment
absent	not mentioned; subjective outcome without blinding of the outcome observer; blinding breakdown confirmed
6) completeness of follow-up	
present	no patients lost to follow-up; acute experimental design does not permit loss of patients; analysis of lost patients provided according to randomization groups, with reason for loss
absent	not mentioned; no analysis of lost patients provided; effect of patient loss to follow-up confirmed
7) allocation concealment	
present	use of consecutive opaque envelopes or pre-ordered treatments; third party assignment of allocation
absent	not mentioned; repeatable pattern of allocation; use of obvious identifiers for allocation (e.g., birth date, record number); assignment of treatment by treating physician

We limited our quality scale to measure criteria that have been demonstrated empirically to be associated with the quality of RCTs. This necessarily excluded many items associated with RCT design and execution that are widely thought to affect quality or that are included in commonly-used quality scales, but it provided us with a defensible "bare minimum" definition of quality. It should be noted that we did not intend our list of criteria to be encompassing of all aspects of quality; our criteria were intended to serve only as a tool for the comparative analysis of the two sets of RCT manuscripts for the purpose of this study.

#### III. Scoring System

Each of the seven criteria was scored as being present (1 point) or as absent (0 points) in the RCT manuscript. Definitions of each criterion are shown in Table 1. If a RCT manuscript did not mention the presence of a criterion, it was considered absent. Conversely, all written statements in the manuscripts were assumed to be accurate both factually and semantically.

#### IV. Criteria Scoring Verification

The intra-rater reliability for the scoring of the quality criteria was determined by comparing the individual criteria scores given to n = 16 RCT manuscripts by one of the authors of this communication (MKB) on two occasions separated by 3 weeks. The correlation coefficient (Kappa) measured in this manner was 0.94.

Inter-rater reliability was determined by comparing the quality criteria scores given to n = 10 RCT manuscripts by two different examiners. One copy of each manuscript was scored by one of the authors of this communication (MKB) while the other copy was scored by an independent examiner (Dr. Babak Jahromi, Department of Neurosurgery, the University of Toronto) who was provided with a thorough description of the criteria. The correlation coefficient (Kappa) for inter-rater reliability was determined to be 0.74.

### Manuscript selection and the screening process

We chose to evaluate the field of brain injury because two search techniques for sampling the population of these RCT manuscripts were readily available. The first search technique was our own PubMed MEDLINE search. The second search technique was performed by the Cochrane Collaboration Injuries (CIG) Group, and forms the CIG trials registry. Copies of the RCT manuscripts identified by these two search techniques were retrieved through the library holdings and interlibrary loan services of five universities.

Next, the manuscripts were read by one of us (CY) to screen-out inappropriately identified manuscripts. Table 2 provides a detailed list of these exclusions. Inherent in the phrase 'randomized controlled trial' is (1) the random allocation of patients into multiple groups for prospective analysis, and (2) the concurrent comparison of at least one group that receives the experimental treatment against another group that does not; manuscripts that did not include random allocation and a concurrent control group were excluded. Furthermore, in order for a manuscript to be considered pertinent to the study of brain injury one of the following conditions had to be met: (1) brain injury had to directly define the patient population; (2) brain injury had to be the cause of a second condition (e.g., seizures) that defined the patient population; or (3) brain injury had to be the outcome measure for the patient population. If none of the above conditions were met the manuscript was discarded from further examination. Duplicate publications, protocol descriptions, abstracts, letters-to-the-editor, and incomplete or preliminary reports were also removed during the screening process.

**Table 2:** Exclusion of manuscripts from the PubMed MEDLINE and CIG Trials Registry groups of manuscripts Manuscripts inappropriately identified by the PubMed MEDLINE search and the CIG trials registry were removed from review during a screening process performed by one of the authors of the current communication (CY).

**reason for exclusion**	**PubMed MEDLINE search**	**CIG Trials Registry group**
INITIALLY IDENTIFIED	*139*	*312*
libraries unable to locate	0	15
unrelated to brain injury	2	3
duplicate publications	2	8
inaccurately claimed to be a controlled trial	22	47
inaccurately claimed to use randomization	11	30
abstracts / letters-to-the-editor	1	31
protocol descriptions	3	0
incomplete / preliminary reports	0	4
non-human subjects	0	1
TOTAL NUMBER DISCARDED	*41*	*139*
REMAINING	*98 *(70% of initially identified)	*173 *(55% of initially identified)

The design and yield of the two search techniques was as follows:

*1) the PubMed MEDLINE search: *The first search technique we used to identify RCT manuscripts pertaining to brain injury involved the PubMed search engine of the MEDLINE database. It was designed to represent a typical literature search performed by a North American researcher who is fluent only in English. The search term "brain injuries" (C10.228.140.199) was used with the limitations of (1) randomized controlled trial, (2) human subjects, and (3) publication in English. The PubMed MEDLINE search included manuscripts indexed from January, 1966, up to February, 2001 (the time at which the search was performed).

         The PubMed MEDLINE search identified n = 139 manuscripts. During the screening process, n = 41          manuscripts from the original 139 (30%) were discarded leaving n = 98 manuscripts (see Table 2 for          a detailed list of the exclusions).

*2.) the CIG trials registry: *The Injuries Group of the Cochrane Collaboration was kind enough to share their list of RCT manuscripts with us for the purpose of conducting this study. The list of manuscripts they provided was compiled by means of the following three steps:

                  step 1) The CIG trials master list was searched using the keywords "head" or "brain" in conjunction                   with "injur*" or "trauma*". The CIG trials master list is a local database maintained at the London                   School of Hygiene and Tropical Medicine that uses a detailed search strategy to identify RCTs                   from multiple computerized databases (a copy of this search strategy is available from Ms. Fiona                   Renton of the London School of Hygiene and Tropical Medicine Fiona.Renton@lshtm.ac.uk) as                   well as various hand searches of journals performed during the writing of systemic reviews; it is                   updated quarterly.

                  step 2) MEDLINE, EMBASE, and CENTRAL databases were searched using the exploded                   keyword "head injuries:ME" or "head injuries:TI". EMBASE includes references from 1974                   onward and, while it uses its own database, it is based on an indexing hierarchy which incorporates                   that used by MEDLINE. Here, MEDLINE was searched with the SilverPlatter search engine, not                   with the PubMed Search engine. Manuscripts of the MEDLINE database indexed as early as 1966                   were accessible to the SilverPlatter search engine. The CENTRAL database is a general list of                   clinical trials that is maintained by the collaborative efforts of multiple Cochrane specialty groups.

                  step 3) Manuscripts identified by hand searches of relevant journals and from references provided                   by direct contact with experts in the field of brain injury were also included.

         The original CIG trials registry was completed in 1998 and was last fully updated in May, 2001; it is that          version which was used in our study.

         The CIG trials registry included n = 312 manuscripts. During the screening process, n = 139 manuscripts          from the original 312 (45%) were discarded leaving n = 173 RCT manuscripts (see Table 2 for a          detailed list of the exclusions).

*3.) overlap between the PubMed MEDLINE search and the CIG trials registry: *Of the total unscreened samples of manuscripts identified through each search technique, n = 80 manuscripts were present in both samples; this corresponded to 58% of the sample of manuscripts identified by PubMed MEDLINE search and 26% of the sample of manuscripts from the CIG trials registry. After the removal of inappropriate manuscripts during the screening process, and scoring process only n = 56 manuscripts were identified by both the PubMed MEDLINE search and the CIG trials registry. This corresponded to 57% and 32% of the PubMed MEDLINE search and the CIG trials registry samples, respectively.

### The scoring process

Each of the RCT manuscripts was read by both authors of the current communication (CY and MKB) who, for clarity's sake, will be referred to as "examiners". One examiner ("non-judging examiner": CY) performed the screening process described previously, then recorded the year-of-publication of each manuscript that survived the screening process in a computerized spreadsheet (Microsoft Excel) and marked them with identification numbers. Then, the non-judging examiner hid the names of the authors of the manuscript, the authors' degrees and departmental affiliations, the journal in which the RCT manuscript was published, and the year-of-publication of the manuscript with black marker. This information was covered wherever it was found in the manuscript so that when the manuscript was scored by the second examiner ("judging examiner": MKB) there would be no potential for bias [8,19]. The data collected by the judging examiner was entered into a computerized spreadsheet that was different from the one linking the year-of-publication of the manuscripts with their identification numbers. The two spreadsheets were combined only when all the manuscripts had been read.

As mentioned above, allocation concealment was included in the list of quality criteria after the first evaluation of the manuscripts. Accordingly, the judging examiner re-read all the manuscripts specifically to determine the inclusion of allocation concealment. The manuscripts were still blinded as described above, and the data was entered into a third spreadsheet that was subsequently analyzed independently of the preexisting data.

Manuscripts in French and Spanish were read without written translation by the judging examiner, whereas written translations were provided to the judging examiner for manuscripts in Japanese (by CY), German and Italian (by Mrs. Margaret K. Borsody), and Chinese (by Language Line, Inc., document translation service).

### Statistical analysis

After completion of the scoring process, statistical analyses were conducted by the judging examiner. The data was considered interval in nature and thus data analysis for discrete variables was used [20]. Furthermore, since this study was constructed as a longitudinal analysis of the change in quality scores over time, it was necessary to use some form of regression analysis to examine the data. Considering these requirements, binary logistic regression analyses were performed for each individual quality criteria. All statistical analyses were done by SPSS (version 11.5, SPSS Inc.). Scores for the individual quality criteria were examined as dependent variables against the independent variable of year-of-publication. Significance is defined as a P < 0.05.

Since the samples of manuscripts from the PubMed MEDLINE search and the CIG trials registry are known to be derived from the same parent population of RCTs (i.e., RCTs in the field of brain injury), it is inappropriate to directly compare them against each other with statistical tests. Rather, it was our goal to analyze the two samples of RCT manuscripts separately, and to make likely conclusions about the parent population from each sample of manuscripts as if there was no other sample of manuscripts available for comparison. Then, knowing that the two samples of RCT manuscripts represent the same parent population, it was our intention to compare the conclusions derived from the separate analyses to determine the impact of the search technique thereupon.

## Results

Regression analysis of the individual quality criteria against the year-of-publication of the RCT manuscripts was performed to determine if the frequency of reporting of each quality criteria changed over time. For the sample of RCT manuscripts identified by the PubMed MEDLINE search, no significant relationship was found for any individual quality criterion (listed in Table 3 with the results from the statistical analysis). The RCT manuscripts identified by the CIG trials registry were also examined in this manner. Analyzing each quality criterion individually as a function of the year-of-publication of the manuscripts in that sample showed that two criteria ("prevention of the movement of patients between groups after allocation, and the use of intention-to-treat analysis"; "the assessment of the distribution of patient characteristics and prognostic factors between groups") and nearly another ("completeness of follow-up") were reported in the manuscripts with increasing frequency over time (Table 4).

**Table 3:** Regression analysis of individual quality criteria versus year-of-publication of the manuscripts identified by the PubMed MEDLINE search This table lists the results of the regression analyses comparing year-of-publication against the individual quality criteria.

**quality criterion**	**regression result** **(W = Wald stat)**	**Cox & Snell R**^2^	**regression line**
the assessment of the distribution of patient characteristics and prognostic factors between groups	W = 0.96, P = 0.33	0.01	y = -0.075x + 9.45
prevention of the movement of patients between groups after allocation, and the use of intention-to-treat analysis	W = 1.63, P = 0.20	0.02	y = 0.088x - 10.30
the blinding of the patients to the treatment they received	W = 0.30, P = 0.58	0.003	y = 0.022x - 0.80
the blinding of the health care providers to the treatments received by the patients	W = 2.00, P = 0.16	0.02	y = 0.055x - 5.71
the blinding of the outcome observer to the treatment received by the patient	W = 0.01, P = 0.93	0.000	y = 0.003x + 0.57
adequacy of follow-up	W = 0.03, P = 0.86	0.000	y = -0.006x + 1.18
allocation concealment	W = 1.06, P = 0.30	0.011	y = 0.39x - 78.4

**Table 4:** Regression analysis of individual quality criteria versus year-of-publication of the manuscripts identified by the CIG Trials Registry This table lists the results of the regression analyses comparing year-of-publication against the individual quality criteria.

**quality criterion**	**regression result** **(W = Wald stat)**	**Cox & Snell R^2^**	**regression line**
the assessment of the distribution of patient characteristics and prognostic factors between groups	W = 5.53, P = 0.02	0.03	y = 0.072x - 4.71
prevention of the movement of patients between groups after allocation, and the use of intention-to-treat analysis	W = 4.74, P = 0.03	0.03	y = 0.054x - 13.25
the blinding of the patients to the treatment they received	W = 0.04, P = 0.84	0.000	y = 0.006x + 0.77
the blinding of the health care providers to the treatments received by the patients	W = 0.16, P = 0.69	0.001	y = -0.010x + 0.32
the blinding of the outcome observer to the treatment received by the patient	W = 0.27, P = 0.60	0.002	y = 0.012x - 0.57
adequacy of follow-up	W = 3.25, P = 0.07	0.02	y = 0.043x - 3.25
allocation concealment	W = 0.23, P = 0.88	0.000	Y = 0.004x - 7.64

## Discussion

Many of reviews have attempted to measure the change in RCT quality over time in a field of medical science. It occurred to us that such an analysis could be influenced by the search technique that was used to identify the RCT manuscripts. Based on this concern we hypothesized that two samples of RCT manuscripts taken from the same field of medical science by different search techniques could provide different measures of the change in quality over time. We empirically tested this hypothesis, and by doing so demonstrated that the conclusions made about the change in quality of RCT manuscripts from a representative field of medical science could be significantly influenced by the search technique that was used to sample the field. This demonstration may then bring into question the validity of previous reviews that have claimed to define the change in quality of RCTs over time in various fields of medical science.

In our study, the samples of RCT manuscripts provided by the PubMed MEDLINE search and the CIG trials registry had less overlap than we would have expected considering that both search techniques involved the MEDLINE database. In particular, the CIG trials registry identified only about 60% of the RCT manuscripts found by the PubMed MEDLINE search despite involving its own search of the MEDLINE database. This observation may ultimately relate to the use of different search terms to identify manuscripts from the MEDLINE database, and to the use of different search engines of the MEDLINE database (i.e., PubMed, versus SilverPlatter in the CIG trials registry) that themselves can affect the identification of manuscripts from the common database. Whatever may be the cause for the discrepancy between our two samples, it may undermine any claim that a search technique necessarily produces a more representative sample from a field of medical science simply because it identifies a greater number of RCT manuscripts.

The two search techniques otherwise differ in several ways. For example, the PubMed MEDLINE search was designed so as to exclude any manuscripts published in a non-English language. This would approximate the typical literature search performed by many researchers in North America, and accordingly all the manuscripts identified by the PubMed MEDLINE search were readily available in local university libraries. Conversely, the CIG trials registry tended to include more references from the non-English language literature (n = 27 manuscripts after the screening process). This inclusiveness of the CIG trials registry seemed to account for the 15 irretrievable manuscripts listed by the CIG trials registry. It is reasonable to state that the non-English language literature is part of medical science and that it should not be discounted solely because of its country-of-origin or the language in which it was written. As another difference, the CIG trials registry involved hand-searches of journals and lists of references provided by authorities in the field of brain injury, which are not features of the PubMed MEDLINE search and which may predispose the CIG trials registry search technique toward recovering more recently-published manuscripts. Recently published manuscripts may be of higher quality, thereby biasing the longitudinal measurement of quality in the RCT manuscript sample provided by the CIG trials registry. Alternatively, such extra efforts would be considered by most to improve on the yield of a search technique by including journals and books that are not indexed by computerized databases.

Arguments can be made that either of the search techniques provided a more representative sample of RCT manuscripts from the field of brain injury, but which search technique is superior – if either can be said to be so – is not a concern of the current study. It was solely our intention to compare the findings provided by two commonly-used search techniques to demonstrate that the search technique can in fact influence the measurement of the change in RCT quality over time. We acknowledge *a priori *that neither of the search techniques we used necessarily sampled RCTs from the field of brain injury in a representative manner. Furthermore, we do not claim to have accurately measured how the quality of RCT manuscripts is changing over time in the field of brain injury with either one of them. This is because we are not confident that either search technique provided a representative sampling of the field of brain injury (i.e., that either search technique had access to all the relevant manuscripts). With regards to the field of brain injury, it is clear from our observations that neither the PubMed MEDLINE search nor the CIG Trials Registry can claim to be complete, as each search technique failed to identify a large number of RCT manuscripts that were found by the other search technique. In other fields of medical science there may be specialized databases or registries that claim to identify *all *relevant manuscripts (e.g., the Renal Registry in the field of nephrology [F.P. Schena, personal communication]). The authors of the current communication cannot understand how such a claim can be made or proven, since demonstrating the completeness of a search strategy would require proving that there are no relevant manuscripts that it does not identify. Ultimately, proving that something does not exist is scientifically impossible. Alternatively, it might be claimed that a search strategy identifies *the majority *of relevant manuscripts. This, of course, depends upon (1) the definition of a majority and (2) the assumption that the finding of even a few relevant manuscripts not identified by the search strategy means there are no other such manuscripts outside of the reach of that search strategy. Again, such a claim would depend upon the assumption that the inability to find further relevant manuscripts indicates that no further relevant manuscripts exist; as described above, this is a scientific impossibility. Rather than claiming perfection or near-perfection, it would seem to us to be more appropriate and accurate to claim that a given search strategy has exhausted all options for identifying relevant manuscripts.

What, then, should be done to avoid a biasing influence related to the search technique during reviews of RCT quality over time? The simplest means of avoiding a such an influence would have apparently been to use multiple search techniques in order to better sample the parent population of RCT manuscripts in a field of medical science. In general, including multiple techniques into a single 'comprehensive' search would be preferable to a simple search involving only a single technique, but even so this does not ensure that the combination is truly comprehensive (as we have demonstrated with the CIG Trials Registry). Essentially this was the goal of the CIG trials registry, but even it did not completely encompass the sample of manuscripts identified by the PubMed MEDLINE search despite involving a MEDLINE search of its own. Similarly, previous reviews of RCT quality have often involved secondary searches following an initial computerized search, but such efforts certainly cannot match the breadth and thoroughness of that from the Cochrane Collaboration. If such reviews of RCT quality are to judge entire fields of medical science it would seem that the search techniques they employ must be shown to produce a representative sampling of the parent population of RCT manuscripts as well as a high yield from that parent population. We hope that the findings presented here bring more attention to this concern in future reviews of the change in RCT quality over time.

## Conclusions

We demonstrated that measuring the change in quality over time of a sample of RCT manuscripts from the field of brain injury can be greatly affected by the search technique. This poorly recognized factor may make measurements of the change in RCT quality over time within a given field of medical science unreliable. The search strategy should be accurately reported in any study that attempts to follow trends in the quality of RCT manuscripts over time, and its limitation in sampling the RCT manuscripts from a field of medical science should be acknowledged and evaluated.

## Competing Interests

The author(s) declare that they have no competing interests.

## Authors' contributions

Both MKB and CY contributed equally to this study, and the exact nature of their contributions are described in the Methods section.

## Pre-publication history

The pre-publication history for this paper can be accessed here:


